# Constitutive Active Mutant TIE2 Induces Enlarged Vascular Lumen Formation with Loss of Apico-basal Polarity and Pericyte Recruitment

**DOI:** 10.1038/s41598-019-48854-2

**Published:** 2019-08-26

**Authors:** Yuqi Cai, Sandra Schrenk, Jillian Goines, George E. Davis, Elisa Boscolo

**Affiliations:** 1 Division of Experimental Hematology and Cancer Biology, Cincinnati Children’s Hospital Medical Center, University of Cincinnati College of Medicine, Cincinnati, OH USA; 2 Department of Pediatrics, University of Cincinnati College of Medicine, Cincinnati, OH USA; 3 Department of Molecular Pharmacology and Physiology, Morsani College of Medicine, University of South Florida, Tampa, FL USA

**Keywords:** Vascular diseases, Mechanisms of disease

## Abstract

Abnormalities in controlling key aspects of angiogenesis including vascular cell migration, lumen formation and vessel maturation are hallmarks of vascular anomalies including venous malformation (VM). Gain-of-function mutations in the tyrosine kinase receptor TIE2 can cause VM and induce a ligand-independent hyperactivation of TIE2. Despite these important findings, the TIE2-dependent mechanisms triggering enlarged vascular lesions are not well understood. Herein we studied TIE2 p.L914F, the most frequent mutation identified in VM patients. We report that endothelial cells harboring a TIE2-L914F mutation display abnormal cell migration due to a loss of front-rear polarity as demonstrated by a non-polarized Golgi apparatus. Utilizing a three-dimensional fibrin-matrix based model we show that TIE2-L914F mutant cells form enlarged lumens mimicking vascular lesions present in VM patients, independently of exogenous growth factors. Moreover, these abnormal vascular channels demonstrate a dysregulated expression pattern of apico-basal polarity markers Podocalyxin and Collagen IV. Furthermore, in this system we recapitulated another pathological feature of VM, the paucity of pericytes around ectatic veins. The presented data emphasize the value of this *in vitro* model as a powerful tool for the discovery of cellular and molecular signals contributing to abnormal vascular development and subsequent identification of novel therapeutic approaches.

## Introduction

Angiogenesis is the process of establishing new blood vessels from the preexisting vasculature. This process is finely controlled by the balance between activators which stimulate blood vessel growth, and inhibitors, which prevent vascular proliferation^1^. Angiogenesis is characterized by a series of temporally distinct events. First, activated endothelial cells (EC) produce proteolytic enzymes (e.g. MT1-MMP) that degrade the extracellular matrix to allow EC to migrate and proliferate to form cellular sprouts and lumens^2^. Also, these cellular protrusions undergo lumen formation by achieving apico-basal membrane polarization^3–6^. Finally, the last step of the angiogenic process is termed blood vessel maturation and consists of deposition of basement membrane and recruitment of pericytes that in turn stabilizes the vasculature while preventing further EC proliferation^1,7–9^.

Improper angiogenic signaling results in malformed vessels that can proliferate excessively generating instability of poorly developed vascular structures with reduced pericyte recruitment. This dysfunctional process is a hallmark of several vascular anomalies and tumor angiogenesis^10,11^. The hyperactivation of tyrosine kinase receptors on EC can lead to pathogenic angiogenesis by driving vascular dysmorphogenesis that resembles human vascular malformations^12–14^.

The endothelial tyrosine kinase receptor TIE2 is a crucial player in the vascular proliferation and maturation processes during angiogenesis by binding to the ligands ANGPT2 (Angiopoietin-2) and ANGPT1 (Angiopoietin-1), respectively.

Activating somatic TIE2 mutations have been identified in EC isolated from lesions of patients with a vascular malformation termed venous malformation (VM)^15–19^. VM manifests as disfiguring painful bluish compressible lesion/s and can affect any tissue or organ. Histologically, VM are characterized by massively enlarged vascular channels with thin EC walls and scant pericyte coverage^18,20^.

VM patient-derived EC and HUVEC (human umbilical vein endothelial cells) engineered to express constitutive active mutant forms of TIE2 (based on mutations found in patients) exhibit increased PI3K (phosphoinositide 3-kinase)/AKT and MAPK (mitogen activated protein kinase)/ERK1/2 signaling^16,21–23^. Hyperactivation of the PI3K and/or MAPK pathway can lead to vascular dysmorphogenesis and are implicated in a multitude of vascular anomalies and vascular tumors^24^.

Injection in mouse of EC expressing constitutive activating TIE2 mutations models enlarged vascular channel formation, recapitulating important aspects of the VM pathology and providing a platform for the testing of candidate targeted treatments^16,18,20,23^. Despite these important findings, the TIE2-dependent mechanisms leading to enlarged lumen formation are still largely unidentified.

Here, we aimed at investigating how a constitutive mutant form of TIE2 modulates some of the fundamental angiogenic steps. We utilized EC expressing TIE2 p.L914F which is the most frequent mutation found in VM patients. We interrogated cell growth, migration speed and polarized movement. We further aimed at generating a 3D (three-dimensional) *in vitro* VM model that could be easily manipulated to recapitulate and decipher fundamental aspects of the aberrant pathological lumen formation. We utilized a fibrin matrix-based system^25^ and investigated lumen size, apico-basal polarity establishment and pericyte recruitment in the TIE2-mutant vascular channels.

## Results

### Constitutive active mutant TIE2 increases wound-induced migration speed with loss of front-rear polarity

**Figure 1:**
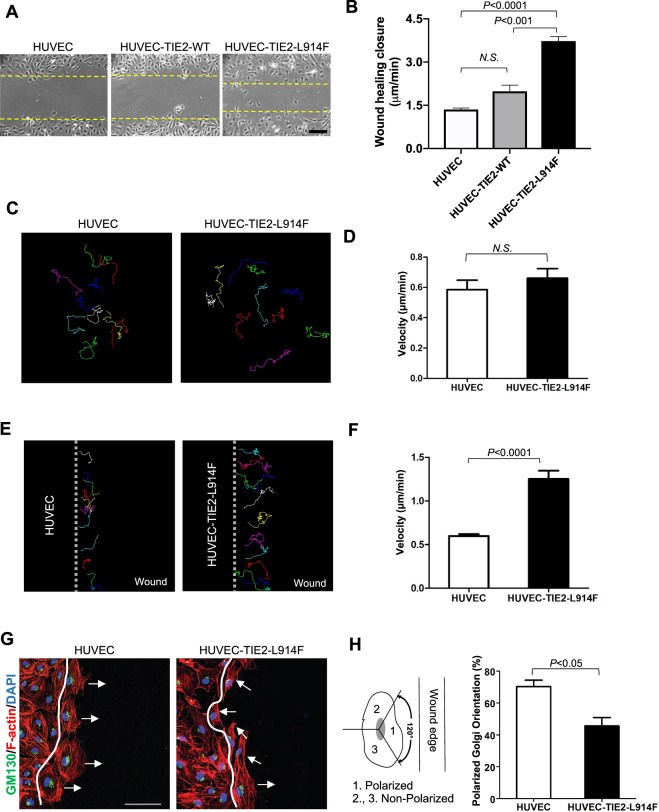
Constitutive active mutant TIE2 increases EC migration in response to wound healing. (**A)** Phase contrast pictures of the wound migration assay of HUVEC (human umbilical vein endothelial cells), HUVEC-TIE2-WT (wild-type) and HUVEC-TIE-L914F, 5 hours after scratching. Dashed lines indicate the wound closure front of migrating EC. Scale bar: 100 μm. (**B)** Quantification of wound healing closure speed (n = 3 independent experiments). (**C)** Analysis of single cell trajectories in non-confluent conditions over a 2-hour time course. (**D)** The cell velocity of 10–12 cells in a non-confluent monolayer was quantified in HUVEC and HUVEC-TIE2-L914F cells (n = 4 independent experiments). (**E)** Analysis of single cell trajectories at the migrating front of a wound healing assay. (**F)** The cell velocity of 10–12 cells at the migration front of a wound healing assay was quantified in HUVEC and HUVEC-TIE2-L914F cells (n = 4 independent experiments). (**G)** Immunofluorescence staining for GM130 (green), Phalloidin (F-actin) (red) and DAPI (blue). Scale bar: 50 μm. (**H**) Quantification of % of cells with polarized Golgi orientation on the moving front of the wound, two hours after scratching (n = 3 independent experiments).

To investigate the angiogenic properties of EC expressing a constitutive active form of the TIE2 receptor we utilized HUVEC engineered to express TIE2-L914F (HUVEC-TIE2-L914F), the most frequent mutation found in VM patients^17,22^. Proliferation and migration are the first events leading to new vessel formation from pre-existing ones^26,27^. HUVEC-TIE2-L914F exhibited growth advantage compared to HUVEC-TIE2-WT (wild-type) and normal HUVEC (Supplemental Fig. S1), as we and others have previously reported^20,28^. Next, we investigated the migration ability of HUVEC-TIE2-L914F compared to HUVEC-TIE2-WT and normal HUVEC and found that HUVEC-TIE2-L914F migrated through a scratch/wound faster than the control cells (Fig. 1A,B). To determine if increased motility is an intrinsic property of HUVEC-TIE2-L914F, we tracked the cell movement trajectories over a 2-hour period. When cells were seeded in monolayer there was no detectable difference between the cell pace of HUVEC-TIE2-L914F and HUVEC (Fig. 1C,D and Supplemental Video 1). Conversely, the migration speed in response to scratch/wound was significantly increased in the TIE2-mutant EC (Fig. 1E,F and Supplemental Video 2). The hallmark of wound migration is re-orientation of the Golgi complex in the direction of the cell migration^29^. To investigate the orientation of the EC during the migration process, EC were fixed 2 hours after performing the scratch/wound and stained with GM130, a marker of the Golgi apparatus^30^. Compared to normal HUVEC that moved perpendicular to the wound, the majority of the HUVEC-TIE2-L914F at the migrating front displayed a non-polarized Golgi apparatus (Fig. 1G,H). These results reveal that expression of the constitutive active mutant TIE2, TIE2-L914F, in EC confers growth advantage and induces migration in aberrant directions due to loss of cellular front-rear polarity.

### HUVEC-TIE2-L914F form massively enlarged VM-like vascular channels in a 3D fibrin gel

**Figure 2:**
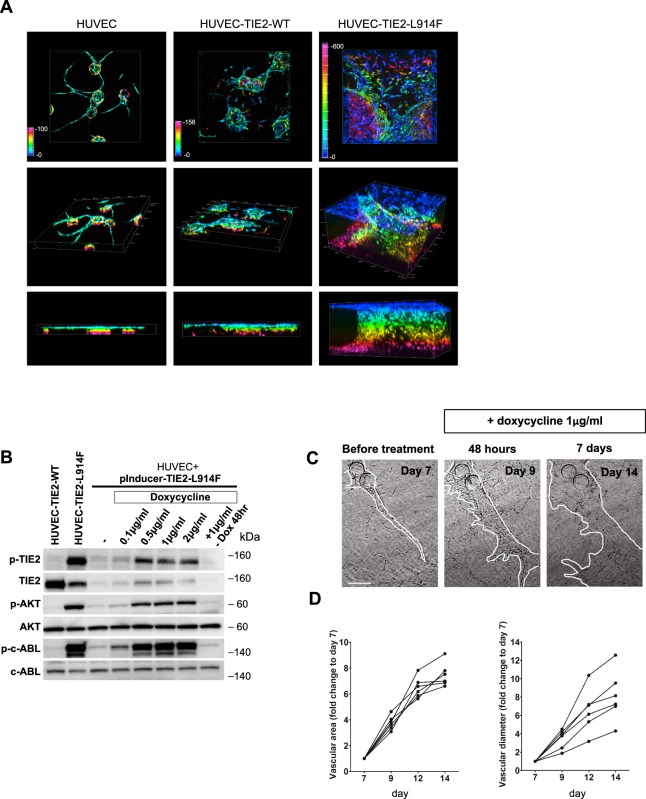
HUVEC expressing constitutive or inducible TIE2-L914F form massively enlarged vascular channels in a 3D fibrin gel. (**A)** Depth color-coded z-stack images (max projection) at day 14 of HUVEC, HUVEC-TIE2-WT and HUVEC-TIE2-L914F in the fibrin gel (top panel), respective images of x-y-z axes (middle panel) and z-y axes (bottom panel). (**B)** Western blot analysis of total cell lysates for the expression and activation levels of TIE2, AKT, c-ABL in HUVEC expressing a doxycycline-inducible form of TIE2-L914F (HUVEC-pInducer21-TIE2-L914F) treated with different concentrations of doxycycline for 48 hours, and control HUVEC-TIE2-WT and HUVEC-TIE2-L914F. (**C)** Phase contrast images of HUVEC-pInducer21-TIE2-L914F 7 days after embedding into the fibrin gel subjected to doxycycline treatment 1 μg/ml and analyzed after 2 (48 hours) and 7 days of continuous doxycycline administration. Scale bar: 100 μm. (**D)** Quantification of vascular area and lumen caliber at different time points after administration of doxycycline. (n = 6 independent vascular fields analyzed for each time point).

When injected *in vivo*, in a xenograft model, HUVEC-TIE2-L914F generated massively enlarged vascular channels, mimicking important aspects of the VM pathogenesis^20,23^. We aimed at generating an *in vitro* 3D (three-dimensional) system as a tool to study VM lumen morphogenesis. When HUVEC were embedded in fibrin gels topped with fibroblasts, they formed regularly shaped lumenized longitudinal vessels (Fig. 2A), as previously reported^25^. Conversely, HUVEC-TIE2-L914F generated ectatic, hollow cyst-like channels that expanded over time invading the gel, while HUVEC-TIE2-WT formed mildly enlarged lumens (Fig. 2A and Supplemental Fig. S2). To further show the effects of the constitutive active TIE2 receptor on the lumen formation, we infected HUVEC with lentivirus expressing a doxycycline-inducible TIE2-L914F (pInducer21-TIE2-L914F). When cells were subjected to doxycycline treatment for 48 hours, they exhibited constitutive TIE2 phosphorylation and activation of downstream effectors AKT and ABL^21^ (Fig. 2B). We embedded the HUVEC-pInducer-TIE2-L914F cells in fibrin gel. Without doxycycline administration, lumens formed as in normal HUVEC. At day 7 of lumen formation, doxycycline 1 μg/ml was added to the gel and analysis at days 9, 12 and 14 (2, 5 and 7 days of doxycycline treatment) revealed lumen/vascular area and diameter enlargement as a result of the TIE2-L914F expression (Fig. 2C,D). These data show that HUVEC-TIE2-L914F in the 3D fibrin gel can phenocopy enlarged lumen formation as detected in VM patients.

### The TIE2-L914F mutation is sufficient to induce ectatic vascular channel formation in absence of fibroblasts

**Figure 3:**
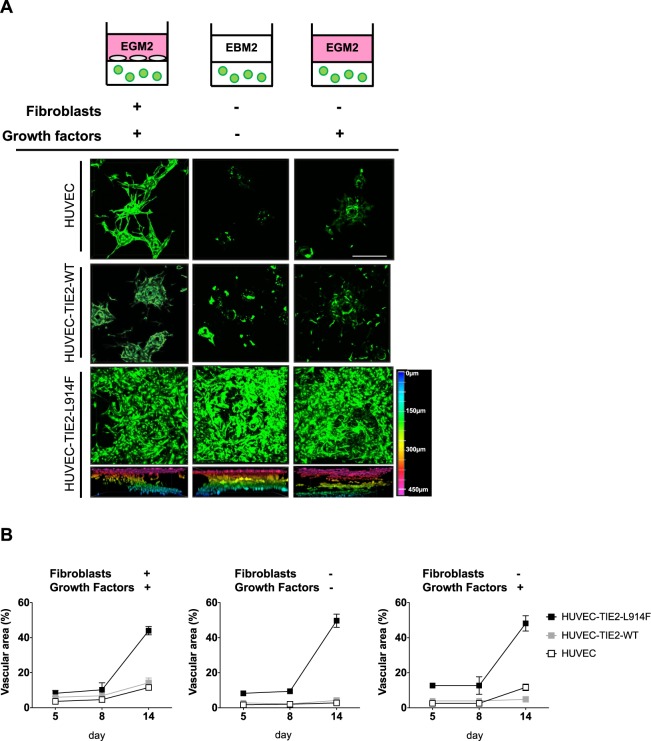
HUVEC-TIE2-L914F form enlarged vascular channels even in the absence of fibroblasts and growth factors. **(A**) Schematic of the experimental conditions utilized (top panel). Z-stack images (max projection) at day 14 of HUVEC, HUVEC-TIE2-WT and HUVEC-TIE2-L914F in the fibrin gel without fibroblasts and/or full growth factor medium (EGM2: endothelial growth factor medium; EBM2: endothelial basal medium). Scale bar: 200 μM. Color-coded diagram of depth images of HUVEC-TIE2-L914F fibrin gel is represented (bottom panel). (**B)** Quantification of vascular area in HUVEC, HUVEC-TIE2-WT and HUVEC-TIE2-L914F fibrin gels with and without fibroblasts and/or full growth factor medium at different time points. (n = 3 independent vascular fields).

In the 3D fibrin gel system employed, fibroblasts are an essential component as they release growth factors that enable EC to sprout and subsequently form lumens^31^. Exogenous growth factors such as ANGPT1 (Angiopoietin-1) and bFGF (basic Fibroblast Growth Factor) cannot rescue the lumen formation in the lack of fibroblasts^31^. To determine the potential of HUVEC-TIE2-L914F to form ectatic lumens in stringent conditions, we generated fibrin gels without fibroblasts and/or growth factors. HUVEC and also HUVEC-TIE2-WT failed to sprout and form lumenized structures in the absence of both fibroblasts and growth factors while HUVEC-TIE2-L914F formed massively enlarged channels (Fig. 3A,B). The addition of growth factors in gels without fibroblasts did not rescue the phenotype in the control EC, while HUVEC-TIE2-L914F ectatic channel formation ability was unperturbed (Fig. 3A,B). The presence of fibroblasts in starvation conditions (no growth factors, EBM2) could still support the sprouting and lumen formation ability of control EC (Supplemental Fig. S3). In our assay, HUVEC-TIE2-WT formed mildly enlarged lumens in presence of fibroblasts and growth factors. Data presented in Fig. 3 suggest this is most likely due to overexpression of the TIE2 receptor, as when fibroblasts are withdrawn these lumens do not form, while HUVEC-TIE2-L914F can still form ectatic channels suggesting the TIE2-L914F mutation is sufficient to drive the phenotype, independently of fibroblast and growth factor stimulation.

### HUVEC-TIE2-L914F generate ectatic vascular channels with loss of membrane apico-basal polarity marker distribution

**Figure 4:**
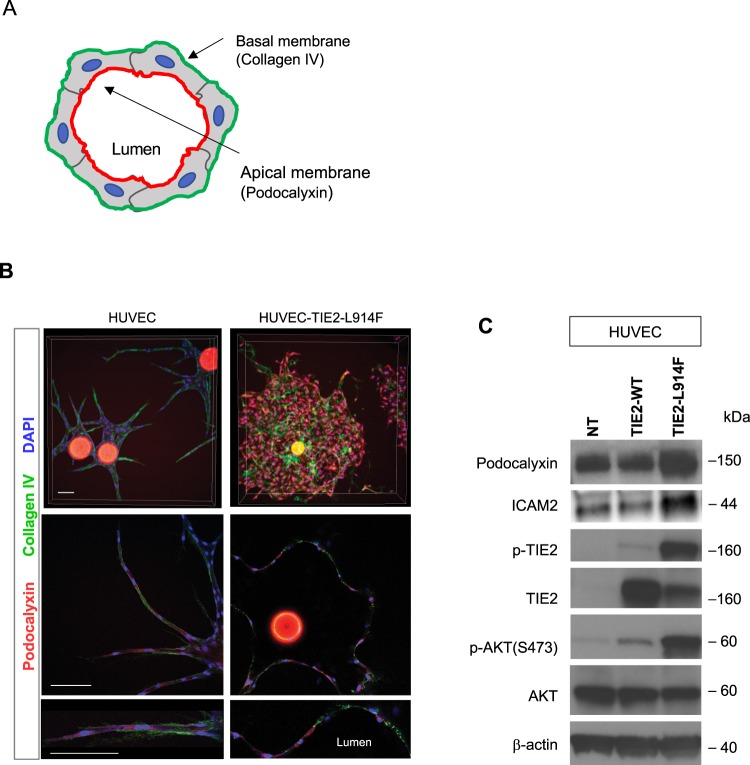
HUVEC-TIE2-L914F generate ectatic vascular channels with disorganized distribution of the apico-basal polarity markers Podocalyxin and Collagen IV. **(A)** Schematic of lumen apical and basal markers, respectively Podocalyxin and Collagen IV. (**B)** HUVEC and HUVEC-TIE2-L914F –derived lumens in the fibrin gel were analyzed for the polarity markers Podocalyxin (apical/luminal marker) and Collagen IV (basal marker). Z-stack images (max projection) (top panels), single slice image taken in the center of the z-stack (middle panel) and zoom-in of single slice to show high magnification details of apical and basal polarity marker distribution (bottom panel). Scale bar: 200 μm. (**C)** Immunoblot analysis for the indicated antibodies of HUVEC, HUVEC-TIE2-WT and HUVEC-TIE2-L914F.

The formation of a vascular lumen requires adequate polarization of the endothelial cell membrane. Apical polarity is defined by the expression of Podocalyxin on the surface of the forming lumen, while the basement membrane marker Collagen IV is localized on the basal side of the EC^32–35^ (Fig. 4A).

To characterize the ectatic vascular channels formed by the HUVEC-TIE2-L914F, we assessed the localization of Podocalyxin and Collagen IV. HUVEC and HUVEC-TIE2-L914F were embedded in fibrin gels and while in normal HUVEC Podocalyxin localizes at the luminal (apical) side and Collagen IV at the basal side, in HUVEC-TIE2-L914F these polarity markers were aberrantly distributed (Figs 4B and Supplemental S4). Expression of the luminal markers Podocalyxin and ICAM2^36^ in the TIE2-mutant EC was increased compared to control EC, HUVEC and HUVEC-TIE2-WT (Figs 4C and Supplemental S5). These data suggest that abnormal establishment of apico-basal polarity during lumen formation and overexpression of Podocalyxin in the mutant-EC may play a role in ectatic lumen formation.

### EC expressing constitutive active mutant TIE2 form channels with scarce recruitment and contact with pericytes, akin to patients’ VM lesions

**Figure 5:**
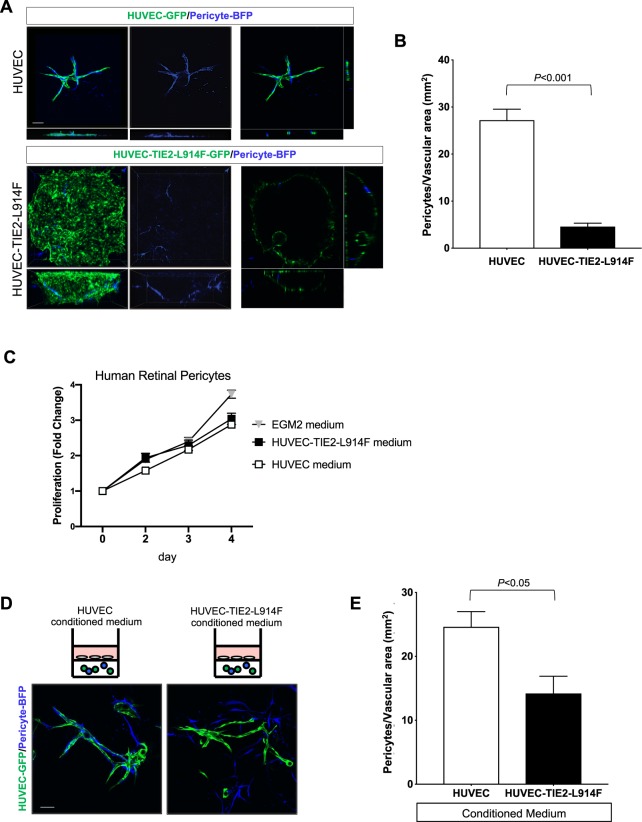
HUVEC-TIE2-L914F form channels with scarce recruitment/contact with pericytes, akin to patients’ VM lesions. (**A)** Z-stack images (max projection) (left panels) of fibrin gels containing GFP-labeled -HUVEC or -HUVEC-TIE2-L914F and BFP (blue fluorescent protein)-labeled human retinal pericytes (10:1 ratio), single slice image taken in the center of the z-stack (right panels) at day 10. Scale bar: 200 μm. (**B)** Quantification of pericytes located on the surface of vascular channels (n = 4–5 independent vascular fields were analyzed). (**C)** Proliferation rates of human retinal pericytes cultured with conditioned medium from HUVEC, or HUVEC-TIE2-L914F, or EGM2 as a normal control culturing condition (n = 3 independent experiments). (**D)** Z-stack images (max projection) of fibrin gels containing GFP-labeled-HUVEC and BFP-pericytes treated with conditioned medium from HUVEC or HUVEC-TIE2-L914F. Scale bar: 200 μm. (**E)** Quantification of pericytes located on the surface of the HUVEC-derived vascular channels (n = 5 independent vascular fields were analyzed).

VM vascular channels are characterized by scarce and irregular pericyte recruitment^18,20,37^. We sought to investigate if the fibrin gel system could mimic this important aspect of the VM aberrant vascular formation. Fibrin gels were assembled with Cytodex® beads coated with a mixture of EC and human retinal pericytes. HUVEC-derived vascular lumens showed significant pericyte coverage, while ectatic channels formed by the HUVEC-TIE2-L914F were contacted only by few, sparse pericytes (*P* < 0.001) (Fig. 5A,B). Next, pericyte cultures were treated for 4 days with conditioned medium from HUVEC or HUVEC-TIE2-L914F, or with EGM2 as control growth medium. There was no difference in the pericyte proliferation curve treated with HUVEC and HUVEC-TIE2-L914F conditioned medium (Fig. 5C), suggesting pericytes’ proliferation is not affected by HUVEC-TIE2-L914F. Next, we investigated if cytokines expressed by HUVEC-TIE2-L914F could affect pericyte coverage in HUVEC-derived vascular lumens. Fibrin gels were set up with Cytodex® beads coated with HUVEC-GFP and pericytes-BFP, and gels were treated with conditioned medium from HUVEC or HUVEC-TIE2-L914F. This experiment revealed a significant difference (*P* < 0.05) in the number of pericytes adhering to HUVEC-derived vascular channels treated with different conditioned medium (Fig. 5D,E).

In summary, this 3D *in vitro* model of VM channel formation recapitulates the paucity of pericytes in the ectatic TIE2 mutant lumens and suggests cytokines released by HUVEC-TIE2-L914F could also influence pericyte coverage in normal, HUVEC-derived vascular structures.

## Discussion

In this study we show that fundamental steps during angiogenesis including migration, lumen formation and maturation are largely aberrant in EC expressing a constitutive active mutant form of the TIE2 receptor. We focused on the mutation TIE2 p.L914F, which is the most frequent mutation found in patients affected by VM. Modeling the VM ectatic lumen formation in a 3D fibrin gel system revealed that the mutation p.L914F is sufficient to induce formation of dilated ectatic channels instead of conventional tubular structures. The HUVEC-TIE2-L914F-derived channels displayed disrupted apico-basal polarity and scant recruitment and contact with pericytes.

HUVEC-TIE2-L914F exhibited faster wound closure migration compared to HUVEC and HUVEC-TIE2-WT. During this process, HUVEC-TIE2-L914F lost the front-rear polarity and migrated in random directions. Conversely, we showed that cell movement in a non-confluent monolayer was similar in mutant and control EC. Previous studies reported that EC expressing the constitutive active TIE2 mutants p.Y897F + p.RL915L exhibit random cell migration movements in monolayer and fail to rearrange in a cobblestone layer, similarly to our results^23^. Other authors reported decreased EC migration in response to VEGF-A (Vascular Endothelial Growth Factor-A) in cells expressing TIE2 p.R849W^38^. The difference in these results could be explained by the fact that p.R849W is a milder activating mutation found in familial cases of VM^18^.

Unpolarized random migration could lead to aberrant lumen formation^4,39^. In VM, vascular lumens are massively enlarged. We modeled the assembly of TIE2 mutant EC into vascular channels akin to VM histology in a 3D morphogenesis assay. While HUVEC organized into a network of tubular structures, HUVEC-TIE2-L914F generated ectatic hollow cyst-like channels. HUVEC-TIE2-L914F generated these dilated vascular structures even in stringent conditions (no fibroblasts and no growth factors) that impeded HUVEC and HUVEC-TIE2-WT from forming sprouts. It is important to note that in this 3D fibrin gel assay HUVEC-TIE2-WT can form mildly enlarged lumens in presence of fibroblasts, we speculate this is due to the overexpression of the TIE2 receptor that responds to the fibroblast and growth factor stimulation. When the cytokines were removed, overexpression of TIE-WT was not sufficient to drive lumen formation. Comparable to our findings, in an EC spheroid sprouting assay, authors reported enlargement of the cell spheroid during a 16-hour period of time when EC expressed the hyperactivating TIE2 mutations: p.Y897F + p.RL915L, p.L914F or p.R849W. HUVEC-TIE2-L914F generate spheroids bigger in size compared to HUVEC-TIE2-R849W^23^, suggesting the levels of TIE2 activation are correlated to lumen size/enlargement.

In both EC and epithelial cells the establishment of apico-basal polarity is necessary for proper lumen formation^40–42^. Apico-basal polarity is regulated by tight and adherens junction organization and is affected by GTPase activity^43,44^. Podocalyxin is a sialomucin that re-localizes in the apical/luminal side of the EC, while basement membrane Collagen IV is a marker expressed in the basal side of the EC^32–35,45^.

In our study, the subcellular localization of these markers followed the above-mentioned pattern in HUVEC-derived lumens but was completely disorganized in HUVEC-TIE2-L914F-vascular channels where Podocalyxin and Collagen IV colocalized in some cells and/or were randomly distributed. A similar phenotype was reported in an *in vitro* model of CCM (Cerebral Cavernous Malformation) based on HUVEC knocked-down for the CCM1/KRIT (Krev-interaction trapped protein 1) gene^34^. CCM is a vascular malformation also characterized by enlarged venous channels^46^. Our data further showed upregulation of Podocalyxin in HUVEC-TIE2-L914F compared to control EC. Germline deletion of *Podxl* in mouse leads to developmental defects and delayed opening of the aortic vascular lumen^5^, while EC-specific deletion results in blood brain barrier disruption during acute inflammation^47^. Podocalyxin overexpression is associated with multiple tumor types and correlates with poor outcome in breast cancer patients^48,49^. In epithelial cells Podocalyxin upregulation is responsible for the recruitment of actin to the apical membrane promoting its expansion^50^. Combined with our data, this suggests that the upregulation of Podocalyxin in the HUVEC-TIE2-L914F has a role in the aberrant apico-basal polarity marker distribution, and that this contributes to the massive lumen expansion.

Pericyte recruitment is essential during angiogenesis to prevent vascular regression and promote vessel maturation^1,7,8^. In VM, the ectatic vascular channels present scarce and irregular perivascular cells^18,28^. When injected in mouse, HUVEC-TIE2-L914F can form enlarged channels lacking pericyte/smooth muscle cell coverage^20^, akin to the human VM. Paucity of perivascular coverage is also found in tumor associated vasculature and in vascular anomalies such as CCM and hereditary hemorrhagic telangiectasia (HHT)^46,51–54^. The lack of Pdgfb (Platelet-derived growth factor b) or its receptor Pdgfrβ in mice result in loss of perivascular cell coverage, with dilated vascular channels and EC hyperplasia^55–58^. In VM, TIE2-mutant EC express low levels of the FOXO1 (Forkhead box O1) target gene PDGF-BB and this could partly explain the decreased pericyte recruitment around the patients’ lesions^28^. Here, we show that even in a system where pericytes are co-seeded with EC on the fibrin gel, pericytes do not move along or migrate to wrap the vascular structures formed by the TIE2-mutant EC, despite HUVEC-TIE2-L914F do not affect the pericyte proliferative ability.

Clinical studies postulate that in VM the lack of pericytes allows for the expansion/dilation of the vascular channels by hemodynamic factors and stretching of the walls of the venous spaces^59,60^. Our data suggest that the expansion of the TIE2-mutated vascular channels is independent of the pericyte contribution as our system does not include flow establishment and HUVEC-TIE2-L914F formed hollow channels that expanded in size with time.

The constitutive activation of the TIE2 receptor induced by the VM patient mutations stimulates both the PI3K/AKT and MAPK/ERK1/2 downstream signaling^16,22,23^. PIK3CA (Phosphatidylinositol-4,5-Bisphosphate 3-Kinase Catalytic Subunit Alpha) gene activating mutations have been found in 20–25% of VM patients^16,22,61^. These mutations result in constitutive active AKT signaling, but do not perturb the MAPK signaling pathway, this would suggest that AKT hyperactivation is sufficient to drive the VM pathogenesis. Of interest, in vascular anomalies a number of mutations in different genes could all be grouped by their downstream effectors being the PI3K and/or MAPK pathway^24^. In a recent study, EC expressing a constitutive active mutant HRAS^V12^ form enlarged sheet-like structures and fail to assemble in elongated tubes in the same 3D fibrin gel assay we utilized^62^. The HRAS-mutant EC phenotype was normalized by a PIK3CA inhibitor BYL719 (Alpelisib) while ERK inhibition result in a failure to undergo lumen morphogenesis and proliferation. This would suggest that in HRAS ^V12^ both pathways can contribute to the phenotype. In VM, future studies are needed to deepen our understanding of the specific contribution of the PI3K and MAPK signaling and to translate this into patient-specific therapeutic options.

In our recently published study, we successfully used this 3D fibrin system to establish the efficacy of mTOR and c-ABL inhibitors in preventing HUVEC-TIE2-L914F derived lumen formation and enlargement^21^. These data further support the use of this system as a tool to study the mechanisms leading to enlarged, ectatic VM lumen and to screen for drugs that could normalize the VM phenotype.

## Materials and Methods

### Cell culture

HUVEC and retrovirally-transfected HUVEC expressing full-length TIE2-WT or TIE2-L914F were a gift from Dr. Lauri Eklund and Miikka Vikkula, and were previously described^20^. All the experiments with human cells were performed in accordance with the Institutional Biosafety Committee guidelines and were approved by the Cincinnati Children’s Hospital Medical Center (CCHMC).

Cells were expanded in culture on 1%(w/v) gelatin/PBS-coated plates and Endothelial Cell Growth Medium (EGM-2) (Lonza) supplemented with growth factors (EGM-2 Single Quot, Lonza) and 10% fetal bovine serum (FBS) (HyClone). Human lung fibroblasts (CCD-19Lu) were purchased from ATCC and cultured in DMEM (Invitrogen)/10%FBS. Human retinal pericytes were purchased from Cell Systems and cultured in DMEM (Invitrogen)/10%FBS. GFP (green fluorescent protein) and BFP (blue fluorescent protein) expressing cells were obtained upon infection with lentivirus expressing pLV-eGFP and pLV-Azurite, respectively (Addgene). Pure eGFP or BFP -expressing populations were obtained upon cell sorting (FACS Core at CCHMC). Doxycyclin inducible HUVEC-TIE2-L914F were obtained upon infection of HUVEC with a lentivirus expressing pInducer21-TIE2-L914F (Addgene).

### Cell migration and movement analysis

For the wound migration assay HUVEC, HUVEC-TIE2-WT and HUVEC-TIE2-L914F were grown to confluence in a six-wells plate in EGM2/10%FBS at 37 °C. Upon reaching confluence the cell monolayer was treated with 2 mM hydroxyurea for 4 hours (to prevent proliferation) and scratch/wounds were performed using a sterile pipette tip. After washing off released cells and cell debris, EBM2/0.5%FBS was added. Time-lapse images were taken over a 5 h period using the Nikon Ti-2 Spectra microscope. The wound healing velocity was calculated after collection of sequential time-lapse images and the relative cell migration distance was measured with ImageJ software (NIH).

For the analysis of individual cell movement, cell velocity in non-confluent conditions (30% confluency) and in response to scratch/wound was measured by imaging with a Nikon Ti-2 SpectraX Inverted Microscope and quantified with Nikon NIS-Elements and Image J. The analysis was performed using sequential time-lapse images over a period of 2 hours. In each wound or non-confluent monolayer 10–12 cells were tracked and all experiments were performed in quadruplicates (n = 4).

### 3D fibrin gel assay

3D fibrin gel assay was performed as previously described^25^. Briefly, Cytodex® 3 microcarrier beads (Sigma) were incubated with HUVEC, HUVEC-TIE2-WT or HUVEC-TIE2-L914F at a concentration of 400 cells/ bead for 4 h at 37 °C. The following day, coated beads were resuspended in 2 mg/mL of fibrinogen (Sigma) solution containing 0.15 U/mL of aprotinin (Sigma) at a concentration of 500 beads/ml. Then 0.625 U/mL of thrombin (Sigma) and 0.5 ml beads/fibrinogen suspension were added per well of a 24-well plate and incubated at 37 °C to allow fibrin clotting. The gels were overlaid with human lung fibroblasts at 2 × 10^4^ cells/well and medium was replaced every other day. Where indicated fibroblasts were omitted and EGM2/10% FBS medium substituted with EBM2 (Endothelial basal medium) which does not contain growth factors. In assays conduced with pericytes the Cytodex® beads were seeded with EC (HUVEC or HUVEC-TIE2-L914F) and pericytes at a 10:1 ratio. Images were acquired with the Nikon A1R LUN-V Inverted Microscope and EVOS cell imaging system (Invitrogen) and analyzed with Nikon NIS-Elements and ImageJ.

### Cell proliferation assay

Cells were seeded at 6000 cells per well in gelatin-coated 96-well plates and cultured in EGM-2 supplemented with 10% FBS. Cell proliferation was measured by sulforhodamine B (SRB) assay^63^ and the optical density (OD) value was read at 540 nm using SpectraMax i3x Multi-Mode Detection Platform (Molecular Devices).

### Immunofluorescence staining

Immunofluorescence staining was performed on cell monolayer, 2 hours after the scratch/wounds was performed. Cells were fixed in 4% paraformaldehyde (Electron Microscopy Sciences) at room temperature for 15 minutes and permeabilized in 0.1% Triton X-100 (Sigma) for 5 minutes. After blocking with 5% horse serum (Vector Laboratories) for 1 hour, cells were incubated with Alexa Fluor® 488 mouse anti-GM130 antibody (12.5 μg/ml; BD Biosciences) or ICAM2 (0.56 μg/ml; Cell Signaling) for 1 hour. Filamentous actin was stained using Phalloidin conjugated to the fluorescent dye tetramethylrhodamine (TRITC) (1:40; Life Technologies). Samples were mounted using Prolong Gold with 4′,6-diamidino-2-phenylindole (DAPI) (Life Technologies) and imaged with a C2 confocal microscope (Nikon).

Whole mount staining was performed on fibrin gels upon overnight fixation in 4% paraformaldehyde at 4 °C. Immunofluorescence was performed using primary antibodies against Podocalyxin (10 μg/ml; R&D Systems), Collagen IV (5 μg/ml; eBioscience) followed by Texas Red and FITC -conjugated secondary antibodies (1:200, Vector Laboratories). Nuclei were stained with DAPI (4′,6-diamidino-2-phenylindole) (1:1000, Life Technologies) and imaged Nikon A1R LUN-V Inverted Microscope.

### Immunoblotting

Cells were washed with PBS then lysed using radioimmunoprecipitation assay (RIPA) buffer (Boston Bioproducts) with protease inhibitor and phosphatase inhibitor cocktail (Roche). The protein concentration was determined using the BCA Protein Assay Kit (Thermo Scientifc). Cell lysates were resolved by SDS-PAGE, transferred to a PVDF membrane (Immobilon-P, Millipore), and then blocked in 5% nonfat dried milk for 1 hour. The membrane was then analyzed by immunoblotting with antibodies against the following: phospho-c-ABL (Y245) (0.4 μg/ml; Cell Signaling), c-ABL (1.5 μg/ml; Cell Signaling), phospho-TIE2 (0.002 μg/ml; Cell Signaling), TIE2 (1 μg/ml; Abcam), phospho-AKT (Ser473) (0.05 μg/ml; Cell Signaling), AKT (0.04 ug/mL; Cell Signaling), Podocalyxin (1 ug/mL; R&D Systems), ICAM2 (0.056 μg/ml; Cell Signaling) and β-Actin (1 μg/ml; Sigma). Membranes were incubated with peroxidase-conjugated secondary antibodies (1:5000 Vector Laboratories). Antigen-antibody complexes were visualized using Immobilon Forte Western HRP Substrate (Millipore) on a ChemiDoc™ Gel Imaging System (Biorad) or chemiluminescent sensitive film.

### Statistical analysis

Data are expressed as mean ± SEM and analyzed by Student’s t-test or parametric one-way Anova after normal distribution and equal variance was assessed. One-way Anova with post hoc (Tukey’s) test was used for multiple comparisons. All calculations were performed using GraphPad Prism. Differences were considered significant for *P* value ≤ 0.05.

## Supplementary information

Supplementary Data (1 of 1)

Supplemental Video S1

Supplemental Video S2

## Data Availability

The authors declare that all data supporting the findings of this study are available within the article and its Supplementary Information Files or from the corresponding author upon reasonable request.
