# Supplementary material for: Constitutive Active Mutant TIE2 Induces Enlarged Vascular Lumen Formation with Loss of Apico-basal Polarity and Pericyte Recruitment
Source: Sci Rep. 2019 Aug 26;9:12352. doi: 10.1038/s41598-019-48854-2 (PMC6710257; doi:10.1038/s41598-019-48854-2)
Supplement: Supplementary file 1 — Supplementary Data (1 of 1) [file 41598_2019_48854_MOESM1_ESM.pdf]

Supplementary Data

Title: Constitutive Active Mutant TIE2 Induces Enlarged Vascular Lumen Formation with Loss of Apico-basal Polarity and Pericyte Recruitment.

Yuqi Cai[1,2], Sandra Schrenk[1,2], Jillian Goines[1,2], George E. Davis[3], Elisa Boscolo[1,2]*

[1]Division of Experimental Hematology and Cancer Biology, Cincinnati Children's Hospital Medical Center and [2]Department of Pediatrics, University of Cincinnati College of Medicine, Cincinnati, OH, USA, [3]Department of Molecular Pharmacology and Physiology, Morsani College of Medicine, University of South Florida, Tampa, FL, USA

*Corresponding author:
Elisa Boscolo, Division of Experimental Hematology, Phone:513-803-7267 Email: elisa.boscolo@cchmc.org
Cincinnati Children's Hospital Medical Center, 3333 Burnet Avenue, Cincinnati, Ohio 45229-3039

Supplemental Figures:

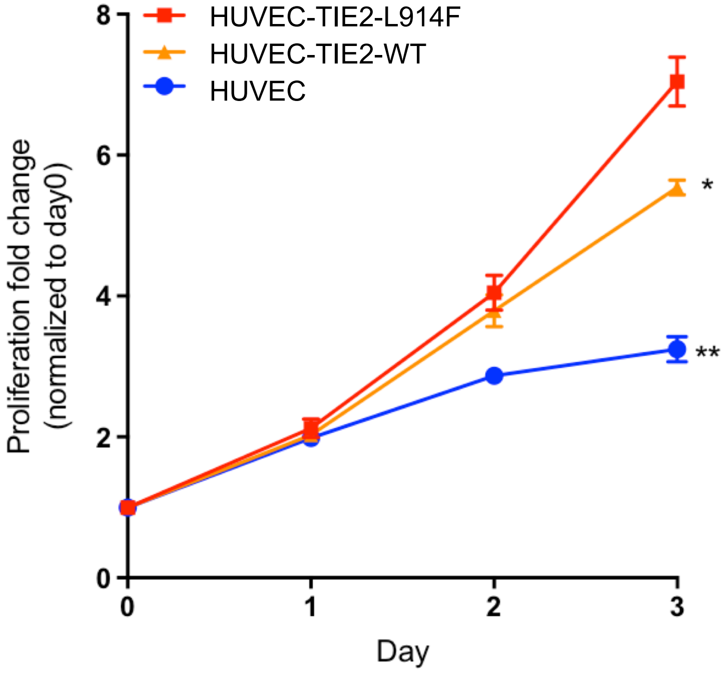

Supplemental Figure S1. Proliferation rate of HUVEC, HUVEC-TIE2-WT and HUVEC-TIE2-L914F grown in EGM2/10%FBS. * $P<0.05$; ** $P<0.001$ at day 3 versus HUVEC-TIE2-L914F

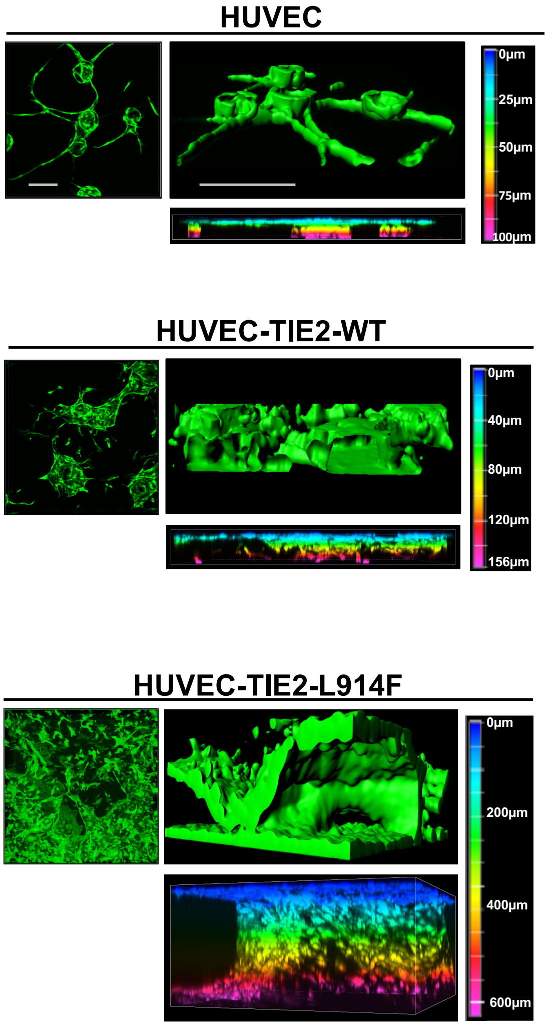

Supplemental Figure S2. Representative 2-dimensional (2D) images, relative 3D structural models and depth color-coded images of vascular channels formed by HUVEC, HUVEC-TIE2-WT and HUVEC-TIE2-L914F. Scale bar 200μm. Adapted from: https://doi.org/10.1161/ATVBAHA.118.312315.

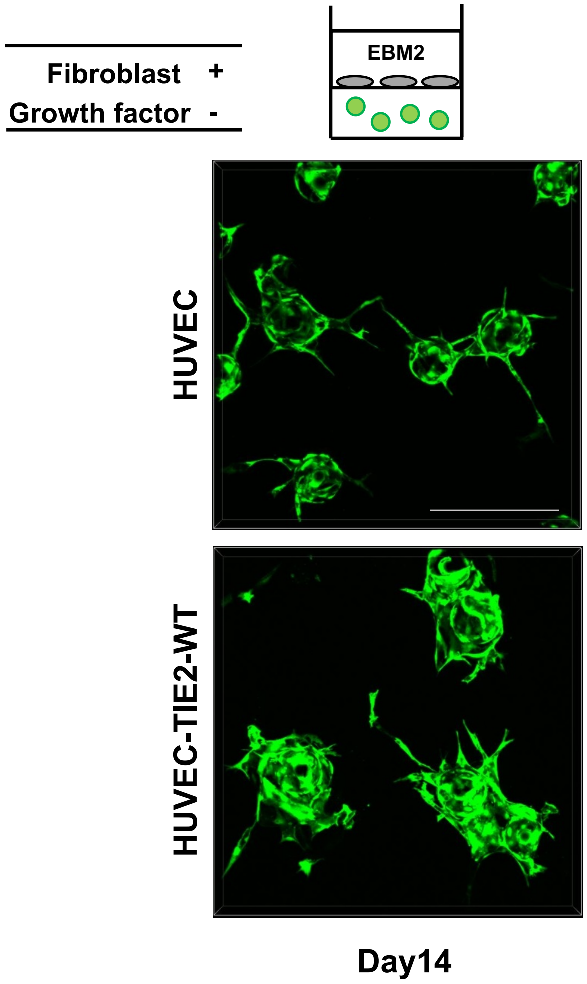

Supplemental Figure S3. Representative z-stack images (max projection) at day 14 of HUVEC and HUVEC-TIE2-WT in the fibrin gel with fibroblasts and without growth factors (EGM2: endothelial growth factor medium; EBM2: endothelial basal medium). Scale bar: 200μM.

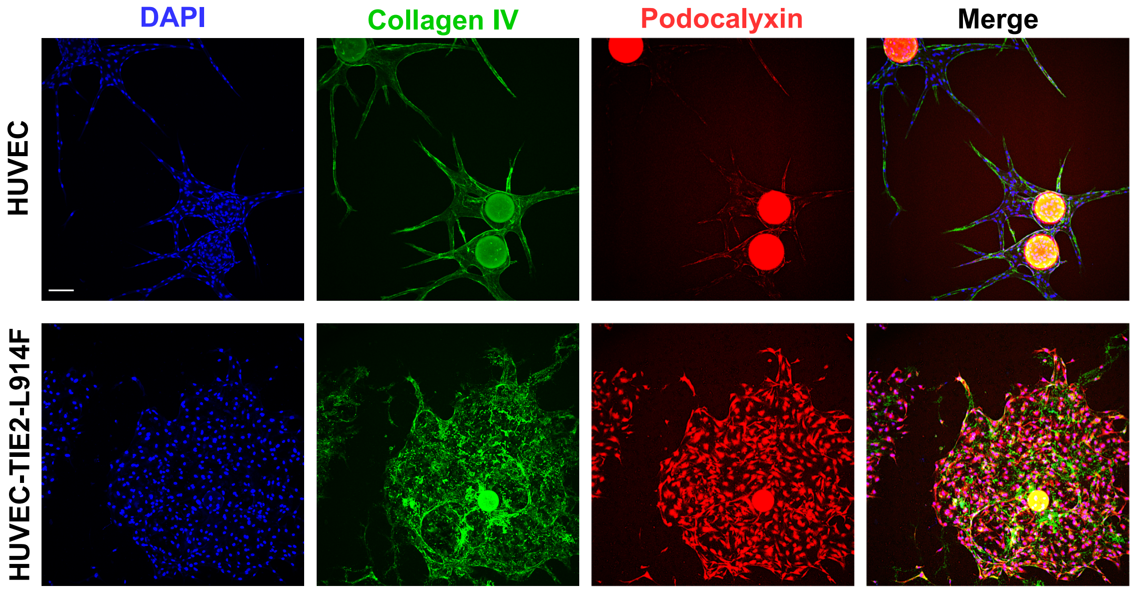

Supplemental Figure S4. Representative z-stack images of HUVEC and HUVEC-TIE2-L914F – derived lumens in the fibrin gel stained for the polarity markers Podocalyxin (apical/luminal marker) and Collagen IV (basal marker), DAPI for nuclei, each single channel and merge. Scale bar: 200µm.

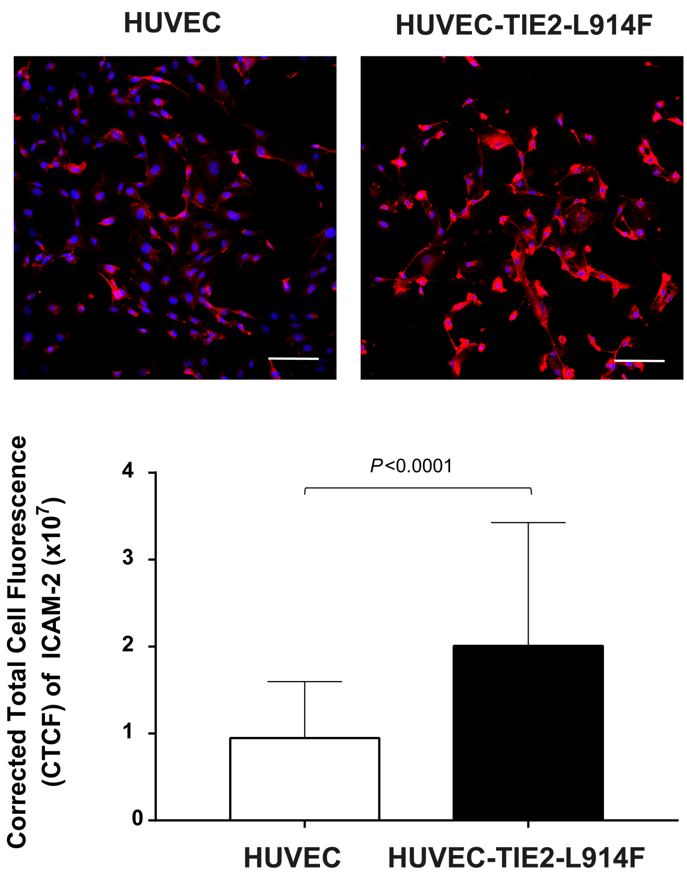

Supplemental Figure S5. Representative images of HUVEC and HUVEC-TIE2-L914F stained for ICAM2 (red) and DAPI for nuclei (blue) (top). Quantification of fluorescence intensity in HUVEC and HUVEC-TIE2-L914F. Scale bar: 200μm.

Supplemental Video 1. Video of free cell movement in monolayer. HUVEC (left) and HUVEC-TIE2-L914F (right).

Supplemental Video 2. Video of cell movement in response to scratch/wound. HUVEC (left) and HUVEC-TIE2-L914F (right).
